# Single Nucleotide Polymorphism (A870G) of the *CCND1* gene: association with colorectal cancer susceptibility 

**Published:** 2017

**Authors:** Mahdi Montazer Haghighi, Mohsen Vahedi, Ehsan Nazemolhosseini Mojarad

**Affiliations:** 1 *Basic and Molecular Epidemiology of Gastrointestinal Disorders Research Center, Research Institute for Gastroenterology and Liver Diseases, Shahid Beheshti University of Medical Sciences, Tehran, Iran. *; 2 *Department of Biostatistics, University of Social Welfare and Rehabilitation Sciences, Tehran, Iran.*; 3 *Gastroenterology and Liver Diseases Research Center, Research Institute for Gastroenterology and Liver Diseases, Shahid Beheshti University of Medical Sciences, Tehran, Iran*

**Keywords:** Colorectal cancer, *CCND1*, Single nucleotide polymorphism

## Abstract

**Aim:**

The aim of this study is to demonstrate the role of *CCND1* gene polymorphism, A870G, in susceptibility to sporadic colorectal cancer in Iranian population.

**Background:**

It has been distinguished that *CCND1* gene is one of the main genes in Wnt signaling pathway which involves in generating colorectal cancer. Nonetheless, there is no consistent result in terms of association between the genetic variations of this gene and colorectal cancer risk.

**Methods:**

We designed a case-control study consisting of 100 subjects with colorectal cancer (CRC) and 100 healthy controls to investigate the effect of A870G polymorphism on CRC susceptibility in an Iranian population. Genotype determination was performed by PCR-RFLP method.

**Results:**

The frequency of GG, AG and AA genotypes for cases were 24%, 51% and 25% respectively, while the genotype frequency for controls were 21%, 50% and 29% respectively. It was identified that there is no significant association between A870G polymorphism and risk of CRC, even after adjusting sex, age and smoking status variables (*P* = 0.777; OR=1.32 95% CI: 0.6-2.93)..

**Conclusion:**

Despite the well-known role of *CCND1* gene in cell cycle regulation, our results revealed that A870G polymorphism could not be a potential predisposing risk factor in genetic susceptibility to CRC, at least in the studied population

## Introduction

 Colorectal cancer (CRC) has a significant proportion burden of cancer morbidity and mortality in the globe (1). It ranks as the fourth leading cause of cancer related death in the world (2). The majority of CRC is attributed to sporadic type which accounts for 80% of whole patients with colorectal cancer. This type obviously illustrates neither familial background nor hereditary pattern.

Basically, uncontrolled cellular proliferation is relevant to cancer development. The cyclin protein family has a promising role in cell proliferation. The cyclin D1 is encoded by the *CCND1* gene which is suited on chromosome11, 11q13. The cyclin protein contributes in regulating of the cell cycle process through the transition cells from G1 to S phase. This process lunches by attaching cyclin D1 to cyclin-dependent kinases (CDKs) 4 and 6 during cell division (3, 4), which consequently leads to release of E2F transcription factor from Rb (tumor suppressor protein). The released E2F transcription factor can effect target genes in downstream in order to prepare the cells for transferring from the G1 phase to the S phase (5). Therefore, it seems that mutations, polymorphisms, amplification and overexpression of cyclin D1 gene might alter cell cycle progression in these steps. These alterations are observed frequently in a variety of tumors and may contribute to tumorigenesis (6).

The G870A as a single nucleotide polymorphism (SNP) of *CCND1* gene has been investigated in different types of cancers, representing its role in promoting the risk of cancers in several populations (7-11). A870G polymorphism (rs9344) in the *CCND1* gene that located in codon 241 (Pro241Pro) involves in the splicing process of the cyclin D1 transcript (12). It is presumed that high level of the cyclin D1 protein associates with an increased risk of cancers (13). The adenine-to-guanine (A/G) substitution at nucleotide 870 (G870A) polymorphism and excessive cyclin D1 activity are common in numerous human tumors such as CRC (14). Although some investigations were conducted to distinguish the possible role of the SNP in colorectal cancer susceptibility, thus far no study has been performed on CCND1 to examine its impact on colorectal cancer risk in Iranian population. Therefore, for further investigation G870A was targeted to study for its likely role in modulating sporadic CRC susceptibility risk in Iranian individuals. 

## Methods

**Sample collection**

Study population of this investigation consisted of 100 individuals who referred to *Research Institute for Gastroenterology and Liver Diseases*, Tehran, Iran, from January 2009 and November 2012, with positive colonoscopy and pathology results for colorectal malignant tumor. All patients voluntarily participated, completed a self-administrated questionnaire and provided peripheral blood samples. Patients that had additionally been diagnosed with hereditary syndrome such as FAP (Familial adenomatous polyposis), HNPCC (hereditary nonpolyposis colorectal cancer) and AFAP (attenuated familial adenomatous polyposis) were excluded from this study. 

One hundred of non-cancer volunteers were selected as control at the same time. The control group contained individuals without personal history or family history of gastrointestinal disorders such as malignancy, polyps or inflammatory ulcers. Controls were matched to cases by sex and age. All participants provided informed written consent prior to participation in this study. Also, all procedures were approved by the ethics committee of the Research Institute for Gastroenterology and Liver Diseases.

**Genotyping**

**Figure 1:**
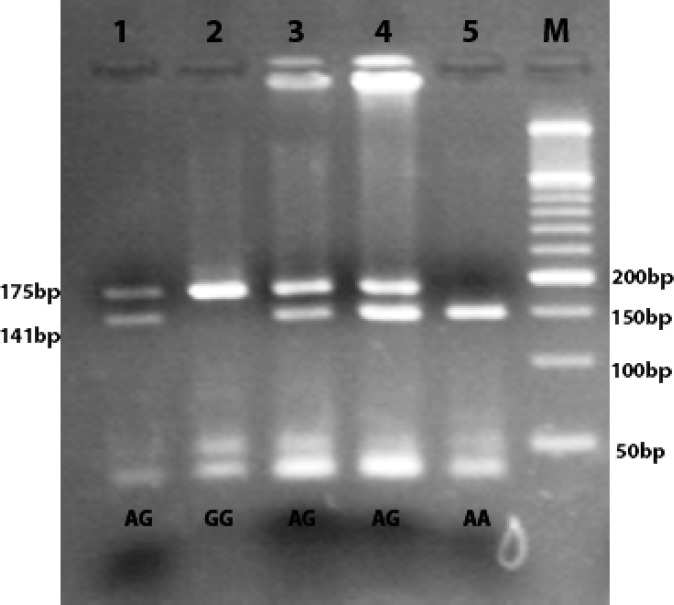
Agarose gel electrophoresis result for three genotypes. Individuals homozygote for the A allele have 1 band at 175 bp (lane 5), those who homozygote for the G allele have 1 band at 141 bp (lane 2) and heterozygous individuals have 2 band at 175 and 141bp (lane 1,3and 4)

Genomic DNA was extracted from 10ml EDTA peripheral blood samples. To identify of CCND1 genotypes, a 212bp fragment including the A870G polymorphism was amplified using Polymerase Chain Reaction-Restriction Fragment Length Polymorphism (PCR-RFLP) method. Primer designed by Gene Runner software version 3.05 (Hastings Software Inc., USA). NCBI Primer-BLAST was used to check primers for specificity. The forward primer 5'-AGTTCATTTCCAATCCGCCC-3'and the reverse primer 5'-TTTCCGTGGCACTAGGTGTC-3' applied to perform PCR. Polymerase chain reaction condition including an initial denaturation of 95˚C for 5 minutes, followed with 35 cycles of 95˚C for 45 seconds, annealing 61.5˚C for 40 seconds, and 72˚C for 45 seconds, with a final extension of 72˚C for 5 minutes. In order to do genotype analysis, the PCR product (212 base pair) were digested with *Msp*I (Moraxella species) restriction enzyme and the digested PCR products were subjected on 3% agarose gel electrophorese (Table 1 and Figure 1). Due to confirming the genotyping results by Agarose gel electrophoresis, 10% of all samples equals ten samples randomly selected and were sequenced by Sanger sequencing. 

**Statistical analysis**

Statistical analysis differences in characteristics between cases and controls were assessed with the chi-square test, as well as, distribution of the allele and genotype was examined using the *χ*^2^ test. Also, to compare the observed genotype frequencies among studied controls and cases with the expected genotype frequencies, we used the *χ*^2^ test to evaluate Hardy-Weinberg equilibrium. Logistic regression analysis was used to adjust the data for probable confounding factors such as age, gender and smoking. Odds ratio (OR) are given with the respective 95% confidence intervals (95% CI). To estimate dominant or recessive effects of the CCND1 A870G genotype on CRC risk, log-likelihood statistics of nested and codominant models were compared. Covariates were identified as potential confounders by examining their distribution by case–control status. The covariates were included in the model if they changed the likelihood ratio statistic (P<0.05) on univariate analysis. 

## Results

Our studied population covered 100 CRC patients and 100 healthy individuals. The characteristics of colorectal cancer cases and controls are represented in Table 2. The cases were compared with controls for following variables: age, sex, and smoking status. The results revealed that the majority of the controls were non-smoking whereas surprisingly the patients demonstrated more smoking habits (12% vs 34%, P<0.001). These results of smoking habits tend to show associated risk with our colorectal cancer patients. The distribution of cases and controls with colorectal cancer by CCND1 genotype are demonstrated in table 3. The frequency of GG, AG and AA genotypes for cases were 24%, 51% and 25% respectively, while the genotype frequency for controls were 21%, 50% and 29% respectively. We provided that there is no significant association between A870G polymorphism and risk of CRC, even after adjustment for sex, age and smoking status (P = 0.777; OR=1.32 95% CI: 0.6-2.93). 

**Table1:** Genotyping fragments length after digested with *Msp*I enzyme

Fragment (bp)	Genotype
141+37+34	AA
175+141+37+34	AG
175+37	GG

**Table 2:** Characteristics of Colorectal Cancer Cases and Controls

Characteristics	Cases(n=100)	Controls(n=100)
Age ,y		
Male, no%	44(44%)	50(50%)
female, no%	56(56%)	50(50%)
Smoking		
No	66(66%)	88(88%)
Yes	34(34%)	12(12%)

**Table 3:** The genotype frequencies of CCND1 A870G polymorphism among CRC patients and controls

Genotype	Patients (n%)	Controls (n%)	OR (95%CI)	P-value
AA	25(25%)	29(29%)	1.00(Ref)	
GG	24(24%)	21(21%)	1.32(0.60-2.93)	0.486
AG	51(51%)	50(50%)	1.18(0.61-2.294)	0.618

CI, confidence interval; OR, odds ratio

**Table 4:** The Allele frequencies of CCND1 A870G polymorphism among CRC patients and controls

Allele	Patients (n%)	Controls (n%)	OR (95%CI)	P-value
A	101(50.5%)	108(54.0%)	1.00(Ref)	
G	99(49.5%)	92(46.0%)	1.15(0.77-1.70)	0.483

The genotype distributions were consistent with Hardy-Weinberg equilibrium in both case and control groups. Also, A allele frequency for the A870G polymorphism was the same among cancer patients as controls (54% vs. 50.5%, P=0.274). There were no significant differences in the genotype frequencies between male and female case subjects. Figure 1 represents the PCR products digested by *Msp*I enzyme (New England Biolabs). The A870G change creates a restriction site for the *Msp*I enzyme with the expected products after digestion with *Msp*I being shown in table 1. As it can be seen in table 4, there are no significant differences in the allelic proportion and it shows no association of increased risk of colorectal cancer and A870G polymorphism.

## Discussion

Colorectal cancer has become a highly prevalent malignancy in recent decades. Effective management of colorectal cancer depends on early detection. Genetic factors are thought to play an important role in the development of colorectal cancer. There are many studies focused on the relationship between genetic polymorphisms and risks of colorectal cancer. It is known that tumorigenesis of colorectal cancer is multi stepped, and cyclin D1 protein may be involved in one of these multiple pathways (10-12). The *CCND1 *is a principle cell cycle regulatory protein which it is often upregulated in human tumors. Furthermore, it is demonstrated that the protein is associated with cell proliferation and poor prognosis.

 A common adenine-to-guanine substitution polymorphism (A870G) at codon 241 in exon 4 of the *CCND1 *gene influences on risk of the early-age onset in several malignant neoplasms, including colorectal cancer (13). It is indicated the polymorphism affects splicing such that exon 5 is not expressed in the A allele. Since exon 5 is involved in rapid turnover, the variant cyclin D1 corresponding to the A allele may have a longer half-life. Thus, it has a potential to cause uncontrolled cell proliferation (14). Many case-control studies reported that patients with *CCND1 *A allele or AA genotype have an increased risk and poor disease outcome in a number of cancer types, such as nasopharyngeal carcinoma and cervical cancer (15-17). Our results are not similar to earlier studies which reported that the A allele and AA genotype have a significant association with cellular proliferation. As far as we know, this is the first study which is conducted in our country in colorectal cancer.

However, numerous studies illustrated opposing results. In some studies, the G allele implicated as a risk allele. In the current study, the GA or AA CCND1 genotypes were not even associated with a borderline increase in the risk of colorectal cancer among patients. 

Our findings for colorectal cancer patients are in contrast with Haber *et al* results, since they found an increased risk of colorectal cancer among individuals with the GA or AA genotype (16). In the present study no association was identified in terms of CCND1 genotypes and the risk of adenoma which was in contrast to a previous study of colorectal adenoma (17). 

Association between the GA or AA genotypes and younger age of onset was reported in hereditary type of colorectal cancer (18). In spite of the likely functional related to the *CCND1 *A870G single nucleotide polymorphism, there is no consistency in published results concerning its association with colorectal cancer. A meta-analysis on the association between A870G polymorphism and the risk of cancer which performed on sixty studies including 18,411 cases and 22,209 controls by Ioannidis *et al* (19) and interestingly, they reported that individuals with homozygous for A allele (AA) had been associated with increased cancer risk significantly (in overall sample, OR 1.23; Caucasians, OR 1.16; and Asians, OR 1.26). It was suggested that the independent small risk associated with the *CCND1 *A870G polymorphism is not useful clinically.

It was also interesting because Bala and Peltomaki were not able to confirm the results which reported by Kong *et al *regarding the dominant allele A was related to an increased age-associated colon cancer risk. Nevertheless, they indicated that the age at onset of colon cancer was lower by 5-6 years in both types of homozygotes (AA and GG) in comparison to heterozygotes (AG) (20-23). 

It seems that the impact of *CCND1 *A870G polymorphism on the risk of colorectal cancer is likely to vary in different racial and ethnic groups with different allelic frequency of the A allele. However, in the first association study between the *CCND1 *A870G polymorphism and colorectal cancer in an Iranian population, the reported frequency A allele, 54% , in an Iranian population was similar to another report which obtained from a Caucasian population (21). In the present study, we did not observe any association between the risk of colorectal cancer and *CCND1 *A870G polymorphism. This finding is in the line with some previous reports on colorectal cancer, neck and head cancer and also the endometrial cancer (22). Bala S* et al. *reported that the AA genotype was relevant to an increased risk of colorectal cancer at younger age and gender (22). Their finding revealed AA variant genotype was associated with a >3-fold increased risk in individuals who were ≤50 years old and females (23). On the other hand, another investigation (24) demonstrated that the effect of AA/AG genotype on colorectal cancer risk was statistically significant for male patients. We could not find any correlation between age and *CCND1 *G870A genotypes nonetheless. The results indicated that there is an association between the mean age of cases and of controls (56.08 vs. 44.9), while, there was no considerable association between gender and genotypes (24-26). 

Our findings revealed that the A allele frequency is slightly higher in our populations than G allele (54% vs.46%). Nevertheless, a significant association was not detected between the genotypes and colorectal cancer risk. In addition, the findings did not demonstrate a trend towards the genotypes being related to risk of colorectal cancer in the population (data did not show). In the studied population variables including smoking, sex accompany with CCND1 genotypes did not show a significant differ in case and control group. Based on the results, there is no significant association between the A870G and risk of sporadic colorectal cancer in Iranian population. Hence, although CCND1 has a principle role in controlling of the cell cycle, the polymorphism in the gene has no association with susceptibility to CRC. Therefore, it seems that the polymorphism is not a promising biomarker for prognosis possible risk of colorectal cancer in our population. However, performing a case-control study with larger sample size is suggested to response more accurately to the question and more in-depth analysis.
